# Investigation of Surface Modification of Polystyrene by a Direct and Remote Atmospheric-Pressure Plasma Jet Treatment

**DOI:** 10.3390/ma13112435

**Published:** 2020-05-26

**Authors:** Alenka Vesel, Gregor Primc

**Affiliations:** Department of Surface Engineering, Jozef Stefan Institute, Jamova Cesta 39, 1000 Ljubljana, Slovenia; gregor.primc@ijs.si

**Keywords:** atmospheric-pressure plasma jet, direct and remote treatment, polystyrene, wettability patterns, mapping, functional groups, XPS

## Abstract

Localized functionalization of polymer surface with an atmospheric-pressure plasma jet was investigated at various treatment conditions. Polystyrene samples were treated with the plasma jet sustained in argon under direct or remote conditions. The two-dimensional evolution of surface wettability and the spot size of the treated area was determined systematically by measuring apparent water contact angles. Modification of surface chemistry and the formation of functional groups were investigated by X-ray photoelectron spectroscopy (XPS). The saturation of surface wettability and functional groups was observed even after a second of treatment providing the sample was placed close to the exhaust of the discharge tube. The spot diameter of the modified area increased logarithmically with increasing treatment time. However, it decreased linearly when increasing the distance. At the edge of the glowing plasma, however, the modification of surface properties was more gradual, so even 30 s of treatment caused marginal effects. With a further increase in the distance from the edge of the glowing plasma, however, there were no further treatment effects. The results are explained by significant axial as well as radial gradients of reactive species, in particular hydroxyl radicals.

## 1. Introduction

The modification of surface properties of polymers has attracted significant attention from the scientific community because of numerous applications. A commonly used technique for tailoring surface properties of polymer materials is treatment by gaseous plasma. Plasma can be sustained at different pressures. Low-pressure plasma is characterized by its uniformity in a large volume, high dissociation fraction of gaseous molecules, and relatively low power density. Opposite to low-pressure plasmas, the atmospheric pressure plasmas are limited to a rather small volume, where a large electric field is present and also the power density is much larger. Atmospheric pressure plasmas are also characterized by large gradients in reactive particles’ density. Apart from reactive plasma species, atmospheric-pressure plasmas may be rather intensive sources of ultraviolet (UV) and/or vacuum ultraviolet (VUV) radiation [1]. Gaseous plasma at atmospheric pressure can be sustained in the air or any other reactive gas, but the preferred configuration employs noble gases. Other reactive gases such as oxygen can be added in small concentrations to enhance the surface chemistry. The key advantage of noble gases is the lack of channels for the loss of the electrons’ energy, such as rotational and vibrational excitation. Apart from elastic processes, the only possible ways of using electron energy are the excitation of metastables, ionization, and excitation of radiative states. On the other hand, electrons in plasmas sustained in molecular gases suffer numerous collisions that result in the loss of their energy, e.g., dissociation and excitation of ro-vibrational states. The molecular radicals are often recombined or relaxed at three-body collisions, which lead to the increased kinetic energy of colliding particles. The kinetic energy is shared with other molecules at collisions; therefore, the gas temperature is increased. Practically the entire discharge power in atmospheric-plasma sources is spent for gas heating. 

The most commonly used atmospheric-pressure plasmas are dielectric-barrier discharges (DBD) and atmospheric-pressure plasma jets (APPJ). DBD plasmas can ensure surface modification of large areas of objects of a simple shape such as foils, whereas APPJ plasmas are better for local treatment of small areas or for treatment of the objects of a more complex shape. Typical diameters of such jets are of the order of several mm, while some authors also reported microjets [2,3,4]. Such a microjet may produce a highly concentrated plasma that causes significant etching of polymers. For example, Guo et al. reported the etching rate of several µm/min [3]. The length of the jet stretching from the discharge tube depends on the gas flow and the surrounding atmosphere, as well as properties of the power supply. Such jets will cause localized surface modification of a polymer sample. The modified surface area will be limited to the spot achieved by reactive gaseous species and UV/VUV radiation. Although several investigations of the surface modification of polymers by atmospheric plasma jets have already been performed [5,6,7,8], only a few papers have investigated in detail the surface finish versus treatment parameters [9,10,11,12].

Nishime et al. investigated the modified area of polyethylene terephthalate exposed to helium APPJ with a small admixture of O_2_ using different treatment conditions [12]. They employed a dielectric barrier discharge for the ignition of plasma streamers, which propagated downstream a flexible plastic tube allowing the treatment of samples at various angles. An additional plasma plume could be sustained at the end of the long flexible tube. The spreading area of the footprint of reactive oxygen species (ROS) on the starch-iodine-agar plates was measured. The spreading area of reactive oxygen species was increasing with increasing exposure time. Significant improvement in the wettability of the polymer was observed. The spot size of improved wettability decreased with an increasing distance from the exhaust of the flexible tube. At a distance of 5 mm, the spot size was approximately 700 mm^2^, which is much more than a spot diameter caused by reactive oxygen species. The substrate holder was either grounded or left at a floating potential. If the sample was at floating conditions, the spot size was smaller for about 30–50% depending on the distance of the sample from the flexible tube. UV and VUV irradiation profiles were also determined. VUV radiation was focused in a very small area of a dimension close to the diameter of the discharge tube. However, a much larger irradiated area was observed for UV radiation. The spot size of the increased wettability on the PET surface was much larger than the footprint of either ROS, UV, or VUV irradiation measured on starch-iodine-agar plates. The area of high hydrophilicity was fairly spherical at incidence angles of 90 and 45°, but it was shortened in one dimension at the incidence angle of 0°, i.e., plasma jet parallel to the sample. 

The results show that VUV photons and reactive oxygen species, such as O atoms, O_3_, O_2_(a), and OH molecules are the key species in APPJ; therefore, it is important to know their fluxes as well as radial and axial gradients of their densities. He/O_2_ APPJ has been well characterized by Ellerweg et al. [13]. Absolute O-atom densities up to about 5 × 10^21^ m^–3^ were observed. In addition, large concentrations of ozone (O_3_) were detected with the maximum value of about 1 × 10^21^ m^–3^. The O-atom density was maximal at 0.6 vol.% of O_2_ in the noble gas, whereas the O_3_ density continued to increase with increasing O_2_ admixture. The concentration of O atoms decreased with increasing distance from the APPJ nozzle, whereas the opposite behavior was observed for O_3_. 

Sousa et al. used a capacitively coupled radiofrequency plasma jet (RF-APPJ) and the APPJ driven by a kHz discharge to investigate the formation of singled delta oxygen O_2_(a) using infrared optical emission spectroscopy [14]. The carrier gas was He with various O_2_ admixtures up to 1 vol.%. Similar dependencies of the O_2_ (a) density versus discharge parameters (i.e., O_2_ admixture, dissipated power) were found for both sources. The absolute density of O_2_(a) was of the order of 10^20^ m^–3^; however, the maximum value found was about 6 × 10^21^ m^–3^. The substantial concentrations of the O_2_ (a) molecules were found even several centimeters downstream of the effluent zone.

Systematic research on the generation of O atoms in the effluent of an APPJ was performed by Reuter et al. [15]. They used an RF-driven discharge sustained either in He or Ar with a small O_2_ admixture. The O-atom density at the nozzle reached the value of approximately 1 × 10^22^ m^–3^ (for He + 0.5 vol.% O_2_). ‘A substantial amount of O atoms persisted even 10 cm away from the nozzle. Unlike other authors, Reuter et al. used a high-power discharge, because they managed to operate the discharge at the power of 150 W. Interesting enough, the gas temperature remained below 80 °C even at the nozzle when the He flux was 2 m^3^∙h^–1^. Reuter et al. also performed modeling of APPJ sustained in Ar with 1 vol.% O_2_ [16]. The O-atom density decreased quite monotonically with the distance from the nozzle. The maximum O-atom density at the nozzle was almost 4 × 10^21^ m^–3^, whereas at the distance of 5 mm it dropped to approximately 1 × 10^21^ m^–3^. 

Properties of time-modulated RF APPJ in Ar with 1 vol.% O_2_ were studied by Jiang et al [17]. The authors found the densities of O atoms and O_3_ of the order of 10^21^ m^–3^. A very high degree of dissociation was observed during each discharge pulse. The signal of O atoms decreased quickly after the pulse, whereas the O_3_ molecules persisted over a microsecond after each pulse. The O-atom density decreased gradually with increasing nozzle distance; however, the opposite trend was observed for the O_3_ density. The presence of a substrate in contact with the plasma jet had an influence on the O-atom density within the jet because of a change in the gas flow pattern causing different air admixing.

Schröter et al. measured the concentration of OH radicals in the humidified He discharge sustained in an RF-driven APPJ [18]. They found an extensive dissociation of H_2_O molecules upon plasma conditions. The density of OH radicals was about 3 × 10^20^ m^–3^. Such a large density expanded several cm along the discharge channel. An order of magnitude lower OH density was observed by Wang et al. [19]. The exact mechanisms of radical kinetics are still not known because of various reactions, including those with metastable Ar atoms [20].

Recently, Golda et al. measured the UV/VUV radiation arising from APPJ sustained in He and Ar [1]. The gas flow rates were just above 1 slm, and a small admixture of O_2_ or N_2_ was added. In the case of Ar feed gas, the Ar_2_^*^ continuum expanded in the range of wavelengths of roughly between 115–135 nm. 

The brief survey of state-of-the-art indicates a rather high degree of dissociation of reactive molecules in plasma sustained in noble gases. Different authors used different configurations, but the concentration of O atoms was roughly about 10^21^ m^–3^. Because APPJ is a rich source of locally confined reactive species, it may be very suitable for the localized functionalization of products. An example is a requirement for enhanced printability of polymeric packaging. In such cases, the printable area expands only on a small segment of the product. Several authors have already reported results on the surface finish of the polymers using APPJ [5,6,7,8,9], and few presented surface mapping or line analyses of induced modification versus the discharge parameters [4,11,12,21]. Recently, we reported systematic research on the surface finish of the polyethylene terephthalate polymer [10]. We found enhanced wettability of this material at prolonged treatment times. The maximum wettability was observed after about 10 s of treatment with APPJ sustained in Ar with traces of water vapor. The distance between the nozzle and the substrate was found to be crucial for the desired surface finish. Although the glowing plasma jet expanded up to 3 cm from the nozzle, the evolution of the surface wettability was observed already for lower distances, whereas, at 4 cm, the effect was marginal even after prolonged treatment times.

In the present paper, we present the results of a comprehensive investigation of the evolution of surface wettability versus distances as well as plasma treatment time for the case of polystyrene. Unlike the prior art, we focused on the time evolution of the spot size of the affected area versus plasma treatment time when the samples were in direct contact with the glowing plasma jet. In another set of experiments, we show the evolution at the edge of the glowing plasma. The evolution of surface wettability and functionalization versus the distance of the sample from the plasma jet is also elaborated. 

## 2. Materials and Methods 

**Figure 1:**
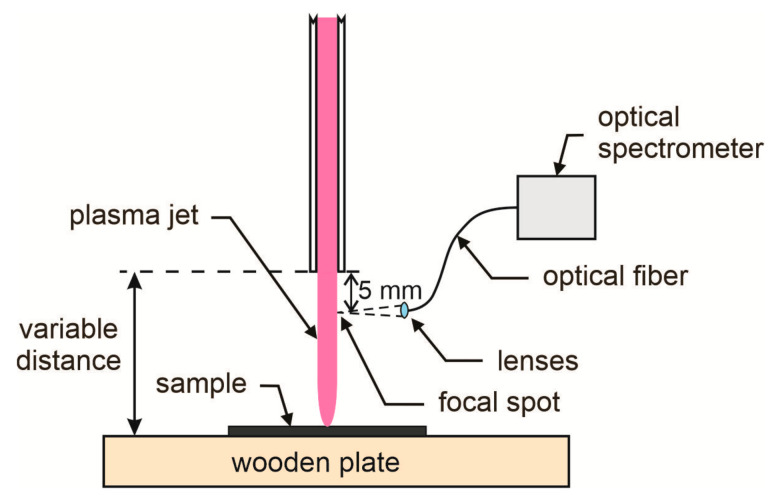
Schematic of the experimental set-up (not to scale).

Polystyrene (PS) foil with a thickness of 125 μm was purchased from Goodfellow Ltd. (Huntingdon, UK). This aromatic polymer contains carbon and hydrogen only with a chemical formula (C_8_H_8_)_n_. Polymer samples were cut to dimensions of 5 × 5 cm^2^ and mounted on to a wooden plate away from any metallic object, so they were at a floating potential upon treatment with gaseous plasma. A single-electrode plasma jet (home-made) was sustained using a frequency of 25 kHz and a peak-to-peak voltage of 7 kV. Ar gas was leaked at a flow rate of 1 slm through a dielectric tube (made from quartz glass) with an inner diameter of 3 mm. The home-made plasma device was installed in a laboratory where the ambient air temperature was 24 °C, and the relative humidity was 65%. The tube was exposed to ambient conditions before performing plasma experiments; therefore, some water molecules that desorb from a dielectric tube can be present in the feed gas. Gaseous plasma was characterized by optical emission spectroscopy. We used AvaSpec-3648 Fiber Optic Spectrometer (Avantes, Apeldoorn, The Netherlands). The spectrometer was connected to the collimation lenses via an optical fiber, as shown in Figure 1. Optical spectra were acquired 5 mm from the nozzle of the APPJ. The schematic of the experimental set-up is shown in Figure 1. 

The samples were treated with the APPJ at various distances from the nozzle ranging from 2 to 45 mm and at various treatment times between 0.5 s and 10 min. After treatment, the samples were probed for wettability with an instrument DSA100 from Kruss GmbH (Hamburg, Germany). The device enabled the automatic deposition of water droplets over the almost arbitrary surface area. For this study, we deposited 73 droplets with a volume of 1 µL on the surface of the samples and acquired the apparent water contact angle (WCA) simultaneously. An apparent water contact angle was measured using a sessile drop method. The droplets were deposited every 5 mm in such a way that the whole surface was mapped. More details about measurements and experimental setup can be found in our previous paper [10].

Some samples were also characterized by X-ray photoelectron spectroscopy (XPS) to investigate chemical modifications. XPS characterization was performed using TFA-XPS spectrometer (Physical Electronics, Münich, Germany). The samples were irradiated with monochromatic Al Kα_1,2_ radiation at 1486.6 eV. The diameter of the analyzed area was approximately 400 µm. Spectra were acquired at an electron take-off angle of 45° in the center of the treated samples. XPS-survey spectra were acquired at a pass-energy of 187 eV using an energy step of 0.4 eV, whereas high-resolution C1s spectra were measured at a pass-energy of 23.5 eV using an energy step of 0.1 eV. An additional electron gun was used to avoid charging of the samples. Spectra were calibrated by setting the C–C peak to 284.8 eV. The MultiPak v8.1c software (v8.1c (2006), Ulvac-Phi Inc., Physical Electronics, Kanagawa, Japan) was used to analyze the spectra. Linear background subtraction was used. The following subpeaks were found in carbon C1s spectra: C–C peak at 284.8 eV, C–O peak at 286.2 eV, C=O and O–C–O peaks at 287,5 eV, O–C=O peak at 288.6 eV as well as O–C(=O)–O peak at 289.7 eV, and the aromatic shake-up peak π - π* at 291.5 eV.

## 3. Results and Discussion

### 3.1. Plasma Characterization

**Figure 2:**
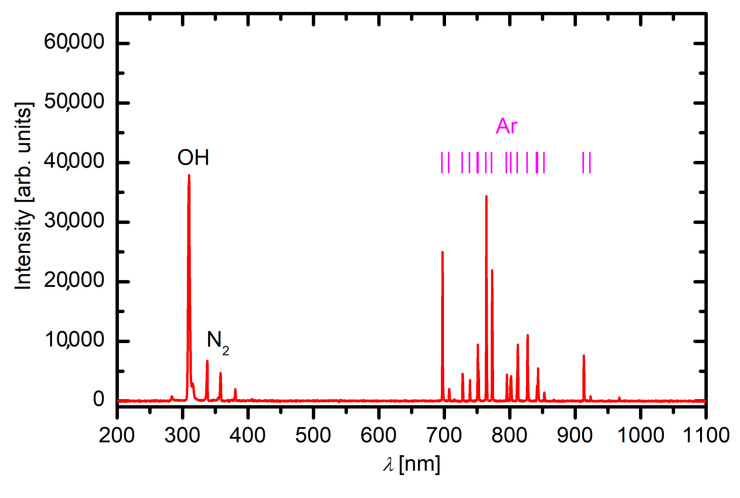
Optical emission spectrum measured at a distance of 5 mm from the nozzle.

Plasma was briefly characterized by optical emission spectroscopy. A typical spectrum is shown in Figure 2. The transitions between highly excited Ar atoms are observed in the red part of the spectrum. These transitions are among the levels of highly excited Ar atoms. Any transition to the ground state occurs at much lower wavelengths (i.e., in VUV range) and could not be probed by our optical spectrometer. According to Golda et al., the major radiation in the VUV range of Ar plasma occurs from the Ar_2_^*^ continuum [1]. The spectrum in Figure 2 also shows a rather intensive OH band with the bandhead at approximately 309 nm. Although water vapor is just an impurity, so the concentration of OH radicals is much lower than the concentration of Ar, the radiation is intensive because of the high excitation probability and good dissociation of water vapor in the discharge. It was reported that the presence of water vapor in Ar plasma suppresses the Ar_2_^*^ continuum [1,22,23]. The addition of such molecular species causes a change in the efficiency of the energy transfer between electrons and heavy particles like water molecules what causes a reduction of electron temperature [22]. Still, radiation in the VUV range of Ar plasma jet is significant [1]. However, the presence of water vapor does not affect only Ar lines. As reported by Nikiforov et al. [22], water vapor will also affect the intensity of oxygen lines much more strongly than Ar lines, because more energy is needed to produce O atoms from H_2_O than to excite Ar. Such weak oxygen lines were also observed by other authors [22,23,24]. As reported by Park et al. [24], oxygen peaks in Ar plasma are very weak even when O_2_ is added to Ar, although Ar/O_2_ plasma is a known source of reactive oxygen species. In our case, a very weak O line at 777 nm was observed only at the shortest distance of 2 mm from the APPJ nozzle, whereas at the distance of 5 mm or more, it was no longer noticeable. Spectrum in Figure 2 also shows weak excited N_2_ lines. As also mentioned by Park et al. weak N_2_ lines can be explained by a lack of exciting agents because ionization and the charge transfer to N_2_^+^ are suppressed in the Ar plasma [24]. Nevertheless, we can conclude that our APPJ is a source of oxidizing species and VUV photons useful for surface functionalization and thus for increasing the wettability of a polymer sample. 

### 3.2. The Effect of the Sample-to-Nozzle Distance

**Figure 3:**
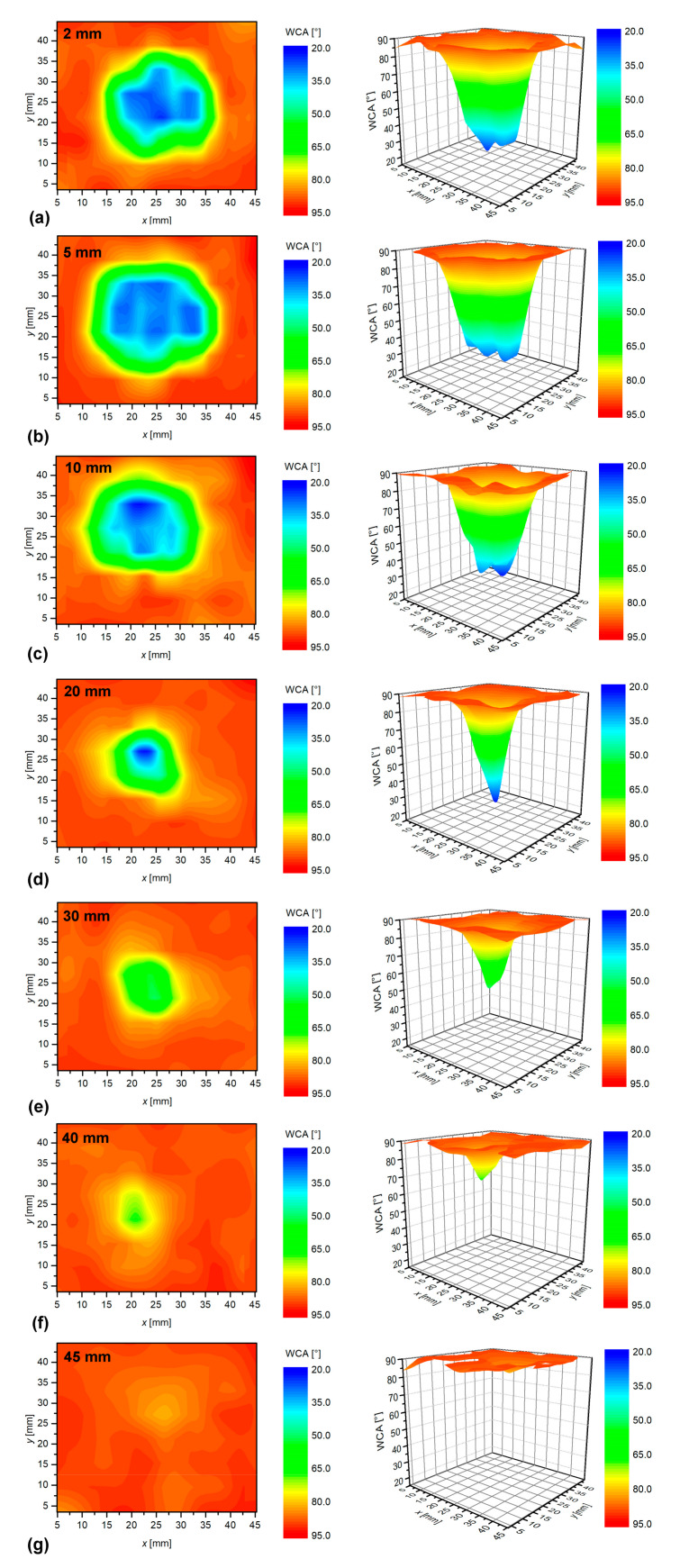
Evolution of the surface wettability for polystyrene treated at various distances from the atmospheric-pressure plasma jets (APPJ) nozzle: (**a**) 2 mm, (**b**) 5 mm, (**c**) 10 mm, (**d**) 20 mm, (**e**) 30 mm, (**f**) 40 mm, (**g**) 45 mm. The treatment time was 30 s.

The 2D/3D mapping of the surface wettability was performed for various treatment conditions. Figure 3 shows the dependence of the surface wettability versus the distance between the nozzle and the sample. A rather large treatment time of 30 s was chosen to make any surface modification visible also for rather large distances between the nozzle and the sample. 

The length of the visible plasma jet was 30 mm. One can observe rather equal effects for distances between 2 and 10 mm (Figure 3a–c). The minimal achievable WCA is about 20°, which is consistent with results reported by other authors. Bradley et al. reported values of approximately 25° for polystyrene treated with a He jet aligned parallel with the surface [25]. Also Dowling et al. reported values between 20–30° using a direct-current pulsed APPJ feed with dry-compressed air [26]. Interestingly, Fricke et al. found values as low as 5° for polystyrene treated with 1.7 MHz APPJ in Ar/O_2_ gas [27]. One possible explanation for obtaining such superhydrophilic surface is the addition of O_2_ and thus a higher concentration of reactive oxygen species that caused better surface functionalization [9]. 

At a distance of 20 mm, however, the hydrophilicity is much less pronounced than at shorter distances. Not only the spot size is much smaller than at shorter distances, but also the optimal wettability is focused to a small spot inside the affected area, as shown in Figure 3d. This effect is even more pronounced with further increasing of the distance between the sample and the nozzle. Figure 3e shows a further shrinkage of the spot size as well as a rather poor wettability even in the center. As mentioned earlier, the distance of 30 mm corresponds to the edge of the glowing plasma. The rapid decrease of the surface wettability is explained by axial gradients of reactive oxygen species. As reported in the introduction, various authors have shown that the O-atom density decreases at least linearly if not more with the distance from the nozzle [13,15,17]. However, for the O_3_ molecules, which are also capable of increasing the wettability of polymers, they found the opposite variation with a distance. Large concentrations of O_3_ were found even far away from the nozzle. The O_3_ molecules definitely form upon the interaction of O atoms with O_2_ molecules, but in our case, no oxygen was added to the discharge zone, so the formation of essential amounts of such rather long-living reactive species is unlikely to occur. The rapid decrease of the surface wettability with increasing distance is therefore explained by a lack of relatively long-living reactive oxygen species capable of functionalization of polystyrene. The effect is even more pronounced at a distance of 40 mm (Figure 3f). Here, the activated area shrinks to the diameter of a few mm only. At a distance of 45 mm, the activation of the polymer is at the edge of the experimental error. Such a rapid decrease also indicates the marginal effect of any UV radiation on the surface finish, as shown by Nishime et al. [12]. They found that UV radiation is decreasing with increasing distance between the plasma jet and the sample. This is even more pronounced for VUV radiation, which has a very limited penetration depth in the surrounding atmosphere in comparison to UV, so it remains much more focused than UV radiation, and at longer distances, it becomes negligible. By considering these effects, we can conclude that the evolution of the wettability versus the distance indicates that the surface is modified by short-living chemical reactive species such as OH radicals. 

**Figure 4:**
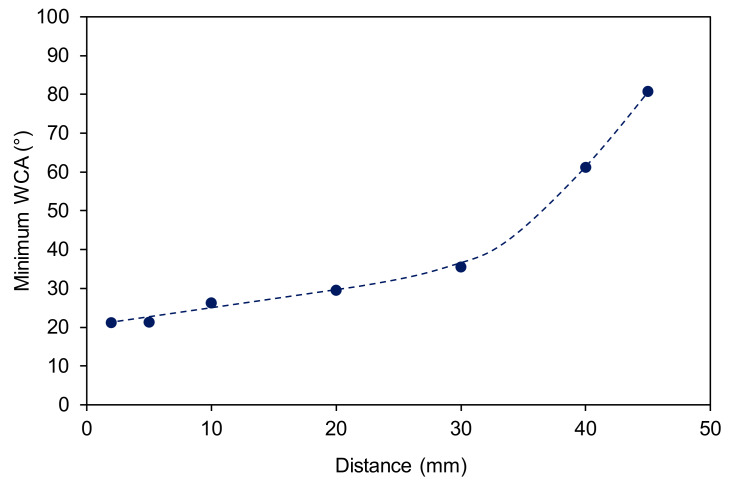
The minimum apparent water contact angle of polystyrene samples treated for 30 s versus the distance.

The images in Figure 4 indicate the surface distribution of apparent water contact angles. The minimum water contact angle is defined as the lowest contact angle of any of the water droplets deposited on the samples’ surface treated at selected conditions. The minimum WCA versus the distance from the sample is shown in Figure 4. One can observe a small increase of the WCA along with the plasma jet until the edge of the visible plasma (about 30 mm). Thereafter, the WCA increases rapidly with increasing distance. The wettability obtained in the center of the affected zone, therefore, does not depend much on the distance as long as the sample is in contact with the glowing plasma, and the treatment time is rather long (surface saturation). 

**Figure 5:**
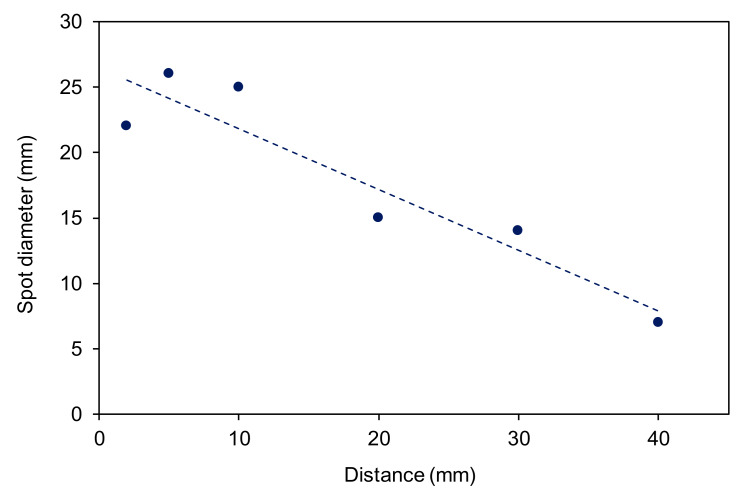
The diameter of the treated zone versus the distance as estimated from Figure 3.

Figure 5 shows the evolution of the diameter of the spot size of the modified area versus the distance between the nozzle and the sample. The results are slightly scattered because it was rather difficult to determine the edge of the affected zone. A yellow ring in Figure 3 was chosen as a border of the affected zone. Nevertheless, the diameter of the spot size decreases with increasing distance. The spot size is always larger than the diameter of the discharge tube, indicating the radial spreading of the reactive gaseous species. 

**Figure 6:**
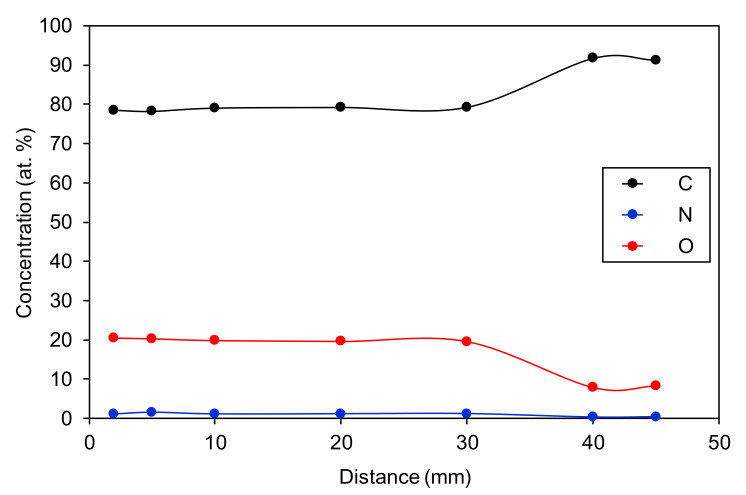
The surface composition of polystyrene measured in the center of the treated zone versus a distance of the APPJ nozzle from the sample surface. The treatment time was 30 s.

The samples treated for 30 s at various distances were also characterized by XPS. Because this technique is time-consuming, we performed characterization only in the center of the affected area. The XPS surface composition is shown in Figure 6. One can see a rather constant concentration of oxygen for distances when the sample was in contact with the glowing plasma, i.e., up to 30 mm. The oxygen concentration is approximately 20 at.%, and minute concentrations of nitrogen were found as well. The appearance of nitrogen within the surface film of polystyrene can be explained by the presence of reactive nitrogen species in the plasma jet because Figure 2 shows some radiation from nitrogen. 

**Figure 7:**
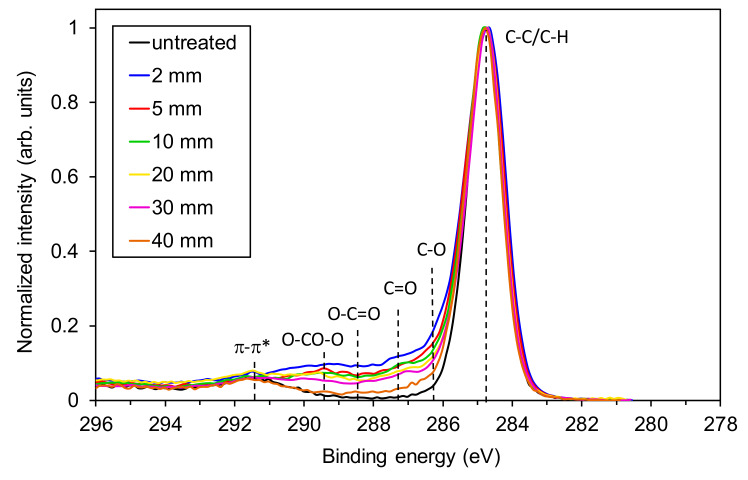
X-ray photoelectron spectroscopy (XPS) C1s spectra of polystyrene samples measured in the center of the treated zone versus a distance of the APPJ nozzle from the sample surface. The treatment time was 30 s.

High-resolution XPS spectra of carbon were also measured for all distances and are shown in Figure 7. Several functional groups typical for plasma-treated polystyrene can be identified, including the highly-polar carboxyl and carbonate groups [9,28,29]. One can observe only a gradual decrease of their intensity (in the range between 286 and 290 eV) for distances between 2 and 30 mm that are corresponding to the visible part of the jet, followed by a more significant decrease for the polystyrene treated at a distance of 40 mm. 

**Figure 8:**
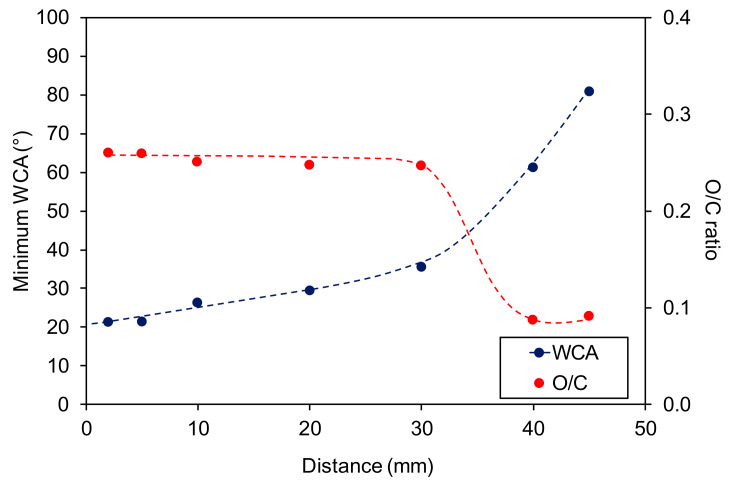
The correlation between the minimum apparent water contact angle and O/C ratio.

There is a fair correlation between the XPS results and the WCA measurements. Figure 8 represents the behavior of the minimum WCA and the O/C ratio versus the distance between the nozzle and the sample. A significant change in both curves appears at the end of the glowing plasma jet, i.e., 30 mm. The chemistry of the afterglow is, therefore, much different from the reactions in the glowing plasma. A feasible explanation of this effect is the short lifetime of species available in our configuration, i.e., predominantly OH radicals and possibly O atoms.

### 3.3. Effect of Treatment Time

**Figure 9:**
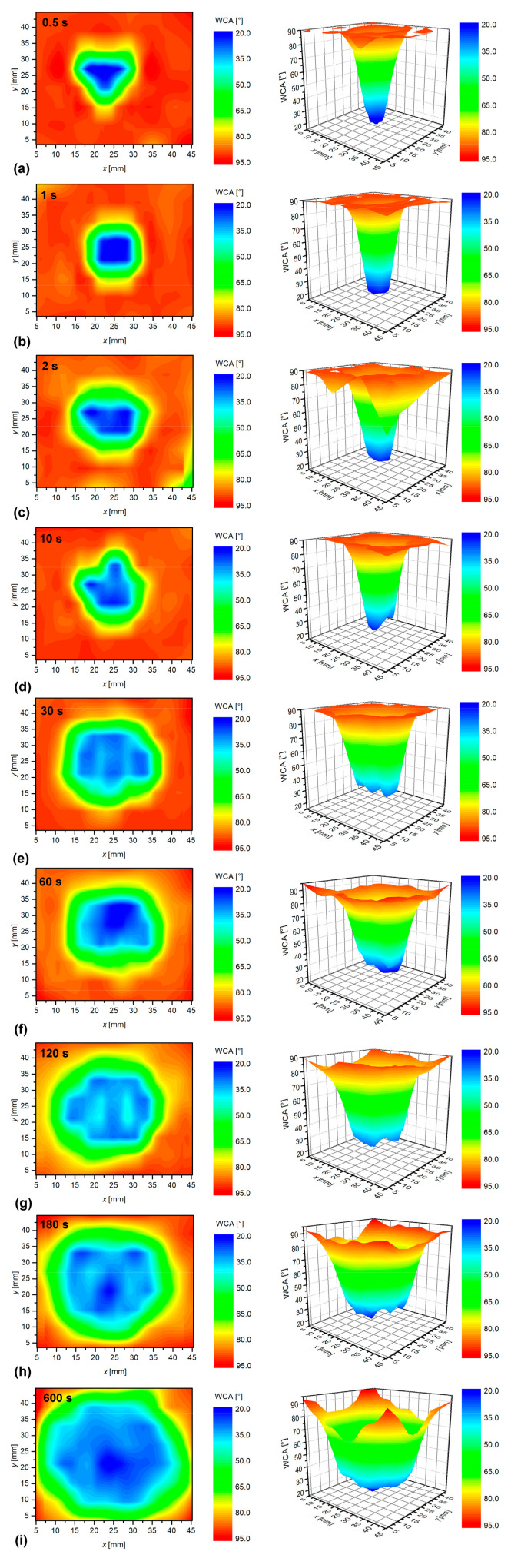
Evolution of the surface wettability for polystyrene treated at various treatment times for the sample distances of 5 mm from the APPJ nozzle: (**a**) 0.5 s, (**b**) 1 s, (**c**) 2 s, (**d**) 10 s, (**e**) 30 s, (**f**) 60 s, (**g**) 120 s, (**h**) 180 s, (**i**) 600 s.

**Figure 10:**
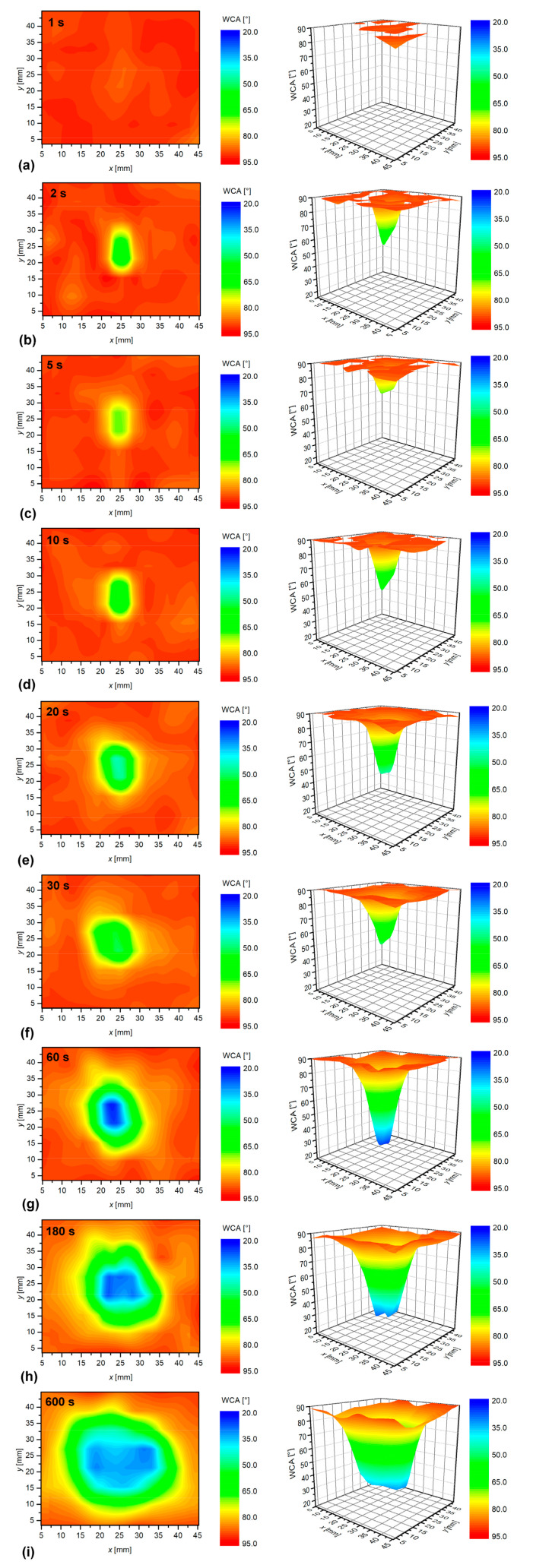
Evolution of the surface wettability for polystyrene treated at various treatment times for the sample distances of 30 mm from the APPJ nozzle: (**a**) 1 s, (**b**) 2 s, (**c**) 5 s, (**d**) 10 s, (**e**) 20 s, (**f**) 30 s, (**g**) 60 s, (**h**) 180 s, (**i**) 600 s.

The temporal evolution of the surface wettability is shown in Figure 9 and Figure 10. Figure 9 was obtained by measuring the 2D distribution of the apparent water contact angle on samples placed 5 mm below the nozzle. Figure 9a reveals a rather good functionalization in the center of the affected area even after half a second of plasma treatment. Such a rapid functionalization is a consequence of a reasonable amount of reactive oxygen species in the glowing plasma, as well as the high affinity of polystyrene for reaction with reactive oxygen species. Previous experiments performed in the low-pressure oxygen afterglows indicated that the O-atom fluence as low as 10^21^ m^–2^ was enough to functionalize polystyrene surface [28]. As shown in the introduction, the density of either oxygen atoms or OH radicals in the APPJ sustained in noble gases easily exceeds 10^20^ m^–2^. Such a density of reactive species will result in the flux over 10^22^ m^–2^∙s^–1^. The fluence of 10^21^ m^–2^ will then be achieved in approximately 0.1 s. The rapid functionalization, as observed in Figure 9a, therefore, indicates an excellent affinity of polystyrene to reactive oxygen species. Here, it is worth mentioning that not all polymers exhibit such properties. E.g., the polyethylene terephthalate polymer was well-activated in the center of the affected zone only after a few seconds of treatment with the same APPJ [10]. 

Prolonging treatment to 5 mm has a little effect on the maximum wettability in the center of the affected zone. In Figure 9b–i, it is clearly shown that the minimal WCA of just above 20° is achieved already upon the shortest treatment time. The changes are observed only in the size of the surface area, revealing the spreading of improved wettability. The area increases monotonously with the increasing treatment time. Finally, after 600 s, almost the entire surface area of 5 × 5 cm^2^ becomes wettable. The effect can be explained by minute concentrations of reactive oxygen species far away from the jet axis, whereas the effect of radiation can be excluded. According to Golda et al., VUV cannot propagate through the ambient air because of strong absorption [1]; therefore, their effect on surface modification away from the axis has to be excluded. This is also in agreement with Nishime et al. [12], who showed that VUV radiation is laterally very limited. This is further supported by our previous paper, where we have used the MgF_2_ optical window to treat the polyethylene terephthalate only with VUV photons, whereas reactive oxygen species were eliminated. The diameter of the area treated with VUV was roughly 10 mm, and it was approximately three-times smaller than without the MgF_2_ window. The spreading of the surface area is, therefore, likely to be a consequence of a small but finite concentration of reactive oxygen species even a few centimeters away from the discharge axis. 

More interesting are the results shown in Figure 10. In this case, polystyrene samples were placed at the end of the glowing plasma jet, i.e., 30 mm from the nozzle. Interestingly enough and opposite to Figure 9b, the WCA remains unchanged even after a second of plasma treatment, as revealed from Figure 10a. A few seconds of plasma treatment causes the development of an affected area, but the area is much smaller than at any experiment performed for the sample position at 5 mm from the nozzle. In fact, the diameter of the spot in Figure 10b–d is not much larger than the diameter of the discharge tube. At such a large distance, the concentration of reactive oxygen species is much lower than at 5 mm, and also, their radial spreading is reduced. There should be strong axial gradients of the reactive oxygen species density in the APPJ used in this study. As reported by Ellerweg et al. [13], Reuter et al. [16] or Jiang et al. [17], the density of short-living radicals like O atoms decreased with increasing distance from the nozzle. According to Reuter et al. [16], the decrease was rather linear, and the O-atom density dropped for an order of magnitude at a distance of approximately 1 cm. By considering these data, one should observe highly wettable surfaces for treatment times one or two orders of magnitude longer than at 5 mm. In fact, Figure 10g indicates that the wettability shows a WCA pattern similar to the one in Figure 9a. The treatment time for Figure 10g was 100 times larger than for Figure 9a. The observations summarized in Figure 9 and Figure 10, therefore, confirm strong axial gradients of plasma radical in our APPJ. 

**Figure 11:**
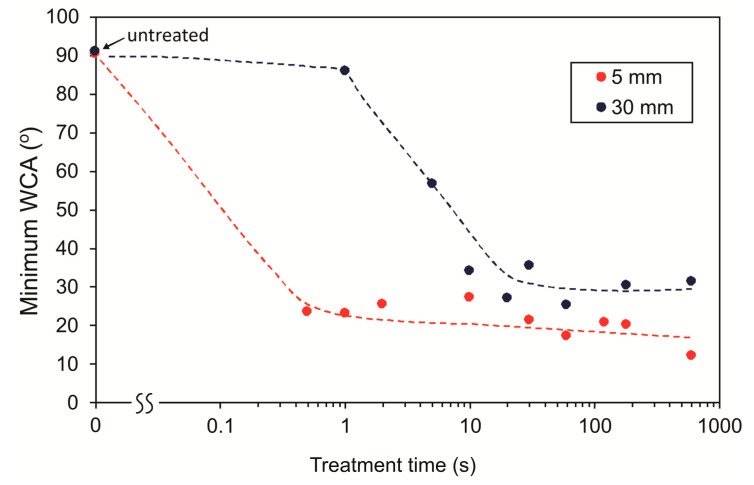
The minimum apparent water contact angle of polystyrene samples exposed to APPJ at distances of 5 and 30 mm versus treatment time.

The minimal WCA in the affected area versus treatment time for both distances is shown in Figure 11. As already observed in Figure 9 and Figure 10, the wettability saturates, and the WCA does not decrease any more after saturation. Saturation is faster for almost two orders of magnitude for the samples treated at a distance of 5 mm. For low-pressure plasmas, it was reported that prolonged plasma treatment may sometimes cause WCA to increase again, which is a consequence of overtreatment that can cause degradation of functional groups and thus loss of enhanced wettability [30]. Highly oxidized functional groups are not stable but tend to decay spontaneously. The decay is much faster at elevated temperatures than at room temperature. As shown in Figure 11, this is not observed in our case. The fact that the minimal WCA does not increase even after 10 min of treatment at the shortest distance indicates a real cold gaseous plasma. As explained already in the introduction, the kinetic temperature of plasma sustained in noble gases remained close to the room temperature because of the lack of superelastic collisions. 

**Figure 12:**
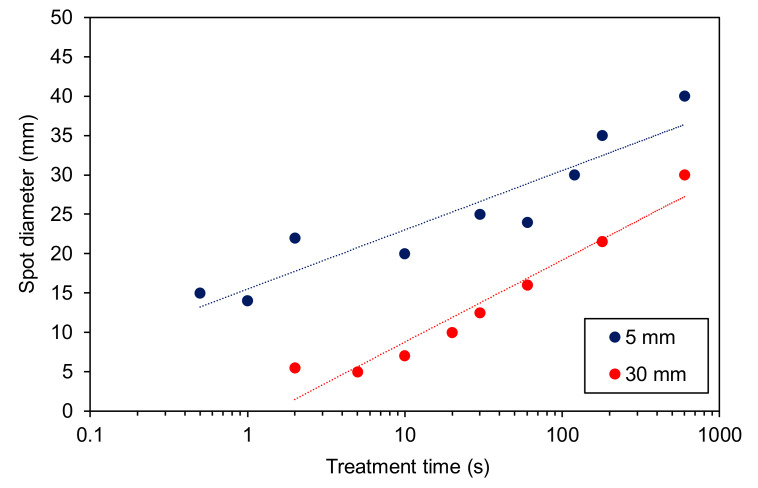
The diameter of the plasma-treated zone on polystyrene samples versus treatment time as estimated from Figure 9 and Figure 10.

Figure 12 represents the diameter of the hydrophilic area versus the treatment time, as estimated from Figure 9 and Figure 10. The diameter is much larger for the samples positioned close to the nozzle, which is explained by the much larger density of reactive species as compared to the edge of the glowing plasma. 

## 4. Conclusions

The evolution of the surface wettability was investigated systematically at various treatment times and distances between the APPJ nozzle and the polystyrene samples. Although no additional reactive gas was leaked into the plasma jet, and only water vapor represented a source of reactive oxygen species, a rather fast functionalization of the polymer surface was observed. Very fast saturation of wettability of polystyrene on the affected area occurred already after 0.5 s of APPJ treatment at the shortest distance of 5 mm. By increasing the distance from the nozzle, the wettability progressed much slower. At a distance of 30 mm, which corresponded to the edge of the glowing plasma, the saturation of wettability was observed for about 100× larger treatment times. The observation is consistent with the gradients of the reactive oxygen species along the axis of the discharge. According to results obtained by other authors, which were presented in the introduction, the density of OH radicals and O atoms decreases sharply along the axis, and only relatively long-living species such as O_3_ are found in the flowing afterglow. In the case of Ar plasma with an admixture of water vapor, the long-living species are almost absent; therefore, the improved wettability of polystyrene at a large distance was observed only upon very long treatment times. The diameter of the affected spot increased rather logarithmically with increasing the treatment time in the range of investigated times. However, when increasing the distance, a linear decrease of the affected spot size was observed. The results of wettability tests performed by measuring the apparent water droplet contact angle are in agreement with the results of functionalization performed by XPS. As the minimum contact angle decreased, the O/C ratio increased. The high-resolution XPS C1s spectra indicates formation of higher concentrations of highly-oxidized carbon groups at lower distances. The initial stage of functionalization, however, cannot be revealed from the results presented in this paper and remains a scientific challenge.

The results summarized in this paper therefore indicate that APPJ can be used for localized treatment of samples; however, the effect of treatment depends on the fluence of reactive species to the surface. Higher doses of reactive species obtained at longer treatment times or lower distances cause not just a better wettability but also larger size of the modified area. Therefore, obtaining very small areas of a high wettability still remains a challenge.
